# Consideration of the Pathological Features of Pediatric Congenital Heart Diseases Which Are Ideally Suitable for Diagnosing With Multidetector-row CT

**DOI:** 10.4021/cr61w

**Published:** 2011-07-25

**Authors:** Yasunobu Hayabuchi, Miki Inoue, Noriko Watanabe, Miho Sakata, Tatsuya Ohnishi, Shoji Kagami

**Affiliations:** a Department of Pediatrics, University of Tokushima, Tokushima, Japan

**Keywords:** Congenital heart disease, Multidetector-row computed tomography, Children

## Abstract

**Background:**

A lots of articles published regarding the usefulness of multidetector-row computed tomography (MDCT) in children with congenital heart disease (CHD) mostly describe that it can be an alternative to the invasive catheterization and angiography. The unique diagnostic features of this imaging modality have been largely ignored or disregarded. We described the pathological conditions that cannot be diagnosed by conventional angiography with cardiac catheterization but can be accurately diagnosed by MDCT.

**Methods:**

We retrospectively reviewed non-ECG-gated MDCT images acquired from 452 children and young adults with CHD between 2005 and 2010 in our institute. In this article, we focused on the diagnostic advantages of MDCT, and indicated five pathological conditions. (1) When Blalock-Taussig shunt total occlusion prevents catheter insertion into the artificial vessel and angiography is ruled out, the peripheral pulmonary artery during the peripheral pulmonary artery can be imaged and diagnosed using MDCT based on blood flow supplied from many small collateral vessels originating from the aorta. (2) The location and protrusion of the device in the vessel after coil embolization to treat patent ductus arteriosus can be accurately visualized by virtual endoscopy using MDCT. (3) Calcification of patches, synthetic blood vessels, and other prostheses that is indistinct on conventional angiograms is clear on MDCT. (4) Simultaneous MDCT observations of the anatomical relationships between arterial and venous systems on the same image can clarify the detail diagnosis for surgical treatment. (5) Compression of the airways by the great vessels and pulmonary segmental emphysematous change can be diagnosed by MDCT.

**Results and Conclusions:**

Among patients with CHD, MDCT is useful not only as a non-invasive alternative to conventional angiography, but also as a tool for specific morphological diagnoses. In the future, it will be necessary to accumulate experience in the recognition of cardiovascular conditions under which MDCT is necessary and to perform as the appropriate examination.

## Introduction

For the clinical management of patients with congenital heart disease (CHD), three-dimensional (3D) accurate evaluation of their morphologic conditions is critical. Advancing multidetector-row computed tomography (MDCT) technology offers opportunities for improved cardiovascular assessment in children [1-3]. One of the many benefits from recent advances is the ability to evaluate in a easier, and much less invasive manner. In light of these advances and its widespread availability, MDCT and 3D imaging postprocessing techniques are increasingly considered to be a quite useful modality for imaging evaluation of cardiovascular lesions in pediatric patients. We have also reported the clinical usefulness of this modality which can be an alternative to invasive angiography, including the measurement pulmonary artery diameter [4], the diagnosis of systemic-to-pulmonary collateral arteries [5], and the presence of Blalock-Taussig (B-T) shunt stenosis [6]. On the other hand, we have been considering that some pathological features in CHD could not be diagnosed by conventional angiography, but accurately depicted only using MDCT. Although there are many reports which indicate the feasibility and usefulness of MDCT for the diagnosis of CHD children, the unique properties which can be available in only MDCT have not been heretofore discussed. This article focuses on the review of unique advantages of MDCT in comparison to other imaging modalities with citing our previous articles [4-13].

## Patients and Methods

### Patients

Subjects comprised 452 consecutive patients with CHD referred to our institution and underwent MDCT between January 2005 and December 2010. The age range of the patients was 0 day to 30 years (mean, 6.8 ± 6.4 years). All protocols were approved by the Institutional Review Board of the Medical University of Tokushima, and written informed consent was obtained from all patients or patients’ parents.

### MDCT Examination

MDCT was performed with patients in the supine position to diagnose and evaluate cardiovascular structure using a 16-slice CT scanner (Aquillion 16; Toshiba Corporation, Medical System Company, Tokyo, Japan).

Scan variables for patients ≥ 5 years old were as follows: collimation, 0.75 mm; pitch, 1.25; effective thickness, 1.0 mm; reconstructive interval, 0.75 mm; voltage, 120 kV; tube current, 150-300 mA; rotation time, 0.50 s; scan time, 8-16 s. For patients < 5 years old, the following scan variables were used: collimation, 0.75 mm; pitch, 1.25; effective thickness, 1.0 mm; reconstructive interval, 0.75 mm; voltage, 100 kV; tube current, 100-150 mA; rotation time, 0.50 s; scan time, 4-8 s. Patients received 2.0 mL/kg of contrast medium [Iopamiron 300 (iopromidol), Nippon Schering, Osaka, Japan] for MDCT angiography intravenously via an antecubital vein using a 22-gauge catheter. In patients with a cannula placed in the dorsum of the hand or wrist, manual injection was performed and saline chaser was used. Scanning was started 10-20 s after the initiation of contrast injection. Sedation was achieved with either 50-100 mg/kg of oral chloral hydrate or 2-6 mg/kg of intravenous pentobarbital. No medication was used to lower or control the heart rate, as is common practice in cardiac imaging of adult patients. Patient heart rates ranged between 55 and 150 beats per minute.

Whilst gating MDCT scans to the cardiac cycle produces significantly fewer motion artefacts than a standard non-gated acquisition protocol [14], non-gated MDCT scans were used in this study. This is because electrocardiogram (ECG)-gated CT angiography is limited by the considerable amount of ionising radiation delivered, degradation of image quality resulting from variations in heart rate and high heart rate, and the strict requirement for patientsto hold their breath during the examination. In addition, a previous study has demonstrated that non-ECG-gated MDCT is usually sufficient for the evaluation of cardiovascular structural abnormalities in patients with CHD [15].

## Results

### Diagnosis of the patency of the pulmonary artery under the total occlusion of Blalock-Taussig shunt

The female patient, who had been diagnosed as heterotaxia, a single right ventricle, pulmonary atresia without central pulmonary artery and systemic-pulmonary collateral arteries, underwent bilateral modified B-T shunt surgery (shunt size 5.0 mm, both) with unifocalization of the collateral arteries to the pulmonary arteries at the age of 3 years. She had occasionally undergone the balloon dilation and stent implantation to counteract shunt stenosis.

**Figure 1:**
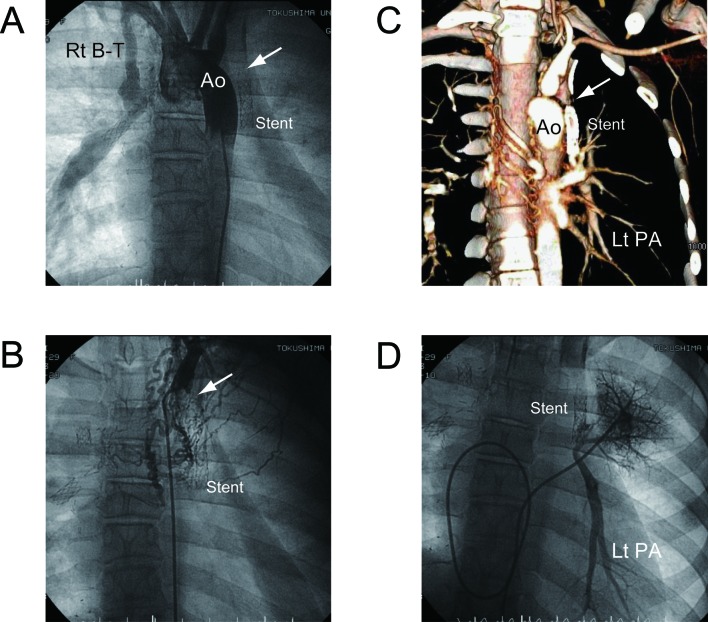
Total occlusion of left modified B-T shunt in a 19-year-old female with a single ventricle. (A) Aortography showing occluded left B-T shunt (arrow) and patent right B-T shunt. (B) Selective left B-T shunt angiography is impossible (arrow). Numerous small torturous collateral arteries originate from left subclavian artery. Patency and caliber of left pulmonary artery is not visualized. (C) MDCT angiography showing occluded B-T shunt (arrow) and patent left pulmonary artery. (D) Pulmonary vein wedge angiography clearly showing left pulmonary artery. Ao, aorta; Rt B-T, right modified B-T shunt; Lt PA, left pulmonary artery. *Reproduced with permission from International Journal of Cardiology 2011, Elsevier limited.*

At the age of 19 years, routine conventional angiography demonstrated complete occlusion of the left B-T shunt and the left pulmonary artery was not enhanced. Neither aortography nor selective arteriography showed the left pulmonary artery (Fig. 1A, B). We attempted to evaluate the left peripheral pulmonary artery by MDCT after the angiography. On MDCT, maintained patency in the left peripheral pulmonary artery was observed despite total occlusion of the B-T shunt (Fig. 1C).

We considered that reconstructing another B-T shunt on the left side can be an available procedure and performed the next cardiac catheterization in conjunction with pulmonary vein wedge angiography. These procedures enabled visualization of the pulmonary artery that was consistent with MDCT images and confirmed the indication for a B-T shunt (Fig. 1D). The next B-T shunt procedure proceeded soon thereafter.

Cardiac catheterization in conjunction with selective angiography remains the gold standard for morphological assessment of B-T shunts and pulmonary arteries, but selective pulmonary angiography is not an option for patients with occluded B-T shunts. The present results show the application of MDCT for demonstrating the patency and caliber of an obscured peripheral pulmonary artery in a patient with a totally occluded B-T shunt [7]. We speculate that numerous small torturous collateral arteries originating from the aorta drain into the left pulmonary artery. Selective angiography is impractical under these conditions, whereas MDCT angiography can yield precise images.

### Evaluation of the location and protrusion of deivce after the coil occlusion of patent ductus arteriosus

We evaluated MDCT images and virtual endoscopy of 10 patients who had undergone the coil occlusion for patent ductus arteriosus [8]. Virtual endoscopy is a compter-generated simulation of endoscopic images derived from MDCT data sets. This technique allows exploration of the inner surfaces of the vessels and its branches [16].

**Figure 2:**
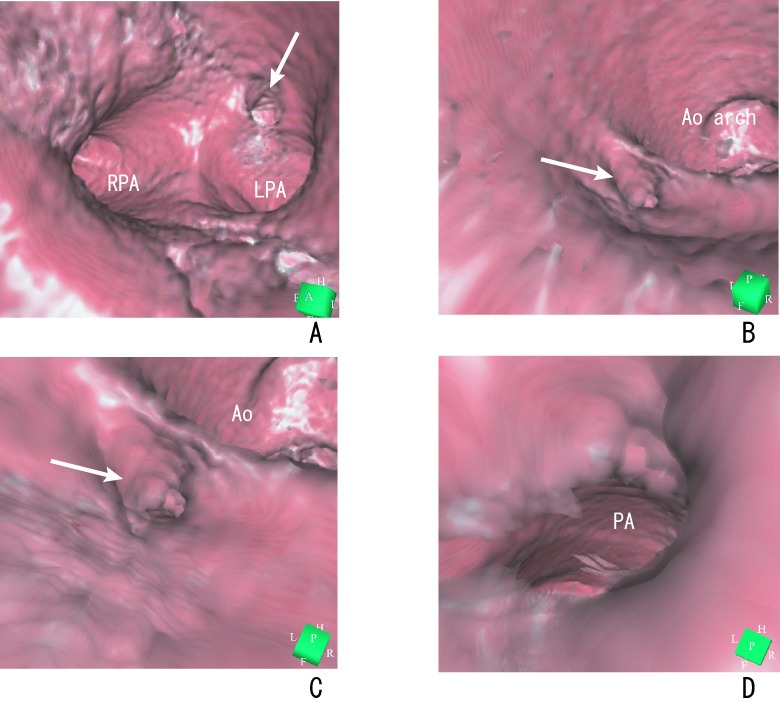
A 13-year-old girl with PDA. (A) Virtual endoscopic image shows the view from the distal main pulmonary artery, with the left pulmonary artery (LPA) and right pulmonary artery (RPA). PDA (arrow) is visible in the main pulmonary artery adjacent to the left pulmonary artery. (B) View from the descending aorta shows the ridge between the aorta and the ductus arteriosus (arrow). (C) View from the descending aorta shows the orifice of the ductus arteriosus and ampulla (arrow). (D) View from the ductal ampulla shows pulmonary artery (PA) visible through the ductus. Ao, aorta; LPA, left pulmonary artery; RPA, right pulmonary artery. *Reproduced with permission from Catheterization and Cardiovascular Interventions 2007; 70: 434-439, Wiley-Liss, Inc.*

Before the coil occlusion, initiating virtual endoscopy in the main pulmonary artery enabled us to identify the left and right pulmonary arteries and the orifice of the PDA. In all patients, we were able to fly through the PDA and continue navigating down into the descending aorta. Navigating from the opposite direction, the virtual endoscopic view from the descending aorta showed that the ductus arteriosus extended from the anterior border of the descending aorta. We could observe the ridge between the aorta and the ductus and the shape of the ductal ampulla, and fly through the PDA into the pulmonary artery (Fig. 2).

**Figure 3:**
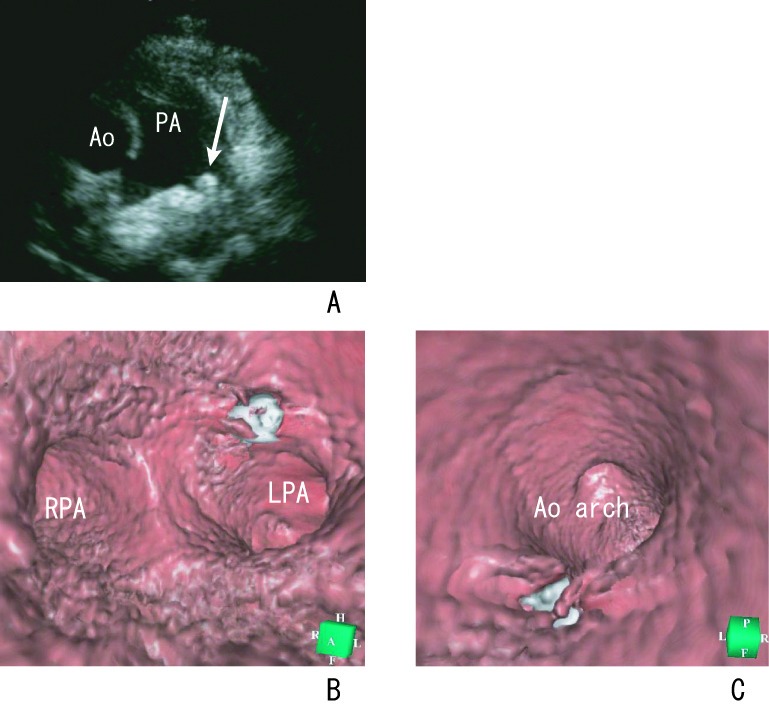
A 6-year-old boy with PDA who underwent transcatheter coil occlusion. (A) Transthoracic echocardiogram shows the coil in the left pulmonary artery. Coil protrusion (arrow) is suspected. Virtual endoscopy shows the location and protrusion of the coil from the pulmonary (B) and aortic (C) sides. The left pulmonary artery is not obstructive. Ao, aorta; LPA, left pulmonary artery; RPA, right pulmonary artery. *Reproduced with permission from Catheterization and Cardiovascular Interventions 2007; 70: 434-439, Wiley-Liss, Inc.*

After coil occlusion, the device protrusion was occasionally visualized into the left pulmonary artery by echocardiography (Fig. 3A). Attempting to define a clinically significant degree of device-induced pulmonary artery stenosis by echocardiography is intrinsically problematic, because device protrusion into the left pulmonary artery is not necessarily accompanied by flow disturbance [17, 18].

Virtual endoscopy depicted the presence and location of the coil from the inside in all patients. Coil protrusion was clearly shown from both the aortic and pulmonary sides (Fig. 3B, C). This depiction was not shown and therefore difficult to assess by echocardiography and conventional angiography. The advantage associated with virtual endoscopy is that it enables evaluation of the inner space of the ductal images. Using this method, we observed the orifice of the ductus and performed a PDA fly-through that provided a virtual view of the catheter approach prior to coil occlusion. Visualization of the coil can also be established by viewing from the inside.

### Calcification of prosthetic patches and synthetic vessels

Polytetrafluoroethylene (PTFE) is a plastic polymer that during the past decade has become popular in the manufacture of synthetic vascular grafts and blood vessel prostheses [19]. However, calcification of PTFE has emerged as an important problem that affects its function and long-term durability [20]. The application of prosthetic PTFE grafts in cardiovascular surgery, particularly in pediatric cardiac surgery, is a widely accepted surgical technique for repair or reconstruction of cardiovascular structures. Recognizing the condition of the postoperative prosthetic graft is important because most patients with repaired congenital heart disease require lifelong cardiac care. Conventional angiography provides minimal information regarding the condition of the patches, synthetic blood vessels, and other prostheses.

**Figure 4:**
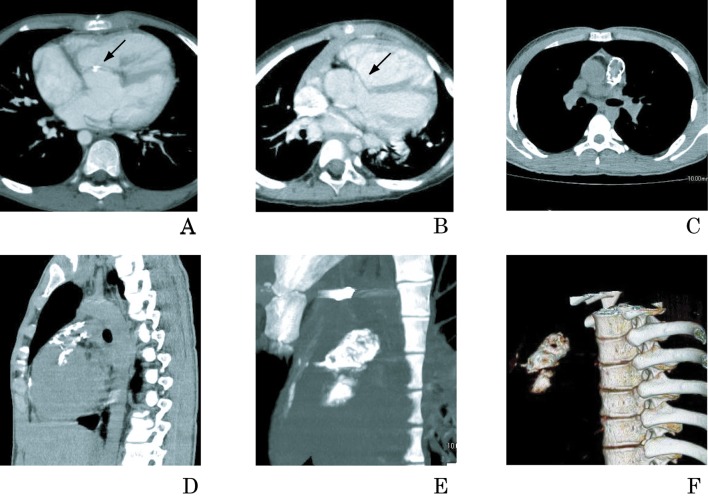
Multidetector-row computed tomographic views of PTFE grafts used for VSD patches and RVOT prostheses. (A) Five-year-old boy with tetralogy of Fallot (TOF) who underwent complete repair with placement of a VSD patch. The axial view reveals spot calcification (arrow) on the VSD PTFE patch. (B) Twelve year-old boy with double outlet right ventricle (DORV) who underwent complete repair with placement of a VSD patch. Multiplanar reconstruction images show VSD patch (arrow) in the axial slice. No calcification is detected. Twenty-year-old man with TOF who underwent complete repair with placement of PTFE graft in RVOT reconstruction. Multiplanar reconstruction images show severe calcification of RVOT in axial (C) and sagittal (D) views. Maximum intensity projection (E) and volume-rendered reconstruction (F) provide spatial and 3D aspects. *Reproduced with permission from American Heart Journal 2007; 153: 806.e1-8, Mosby, Inc.*

**Figure 5:**
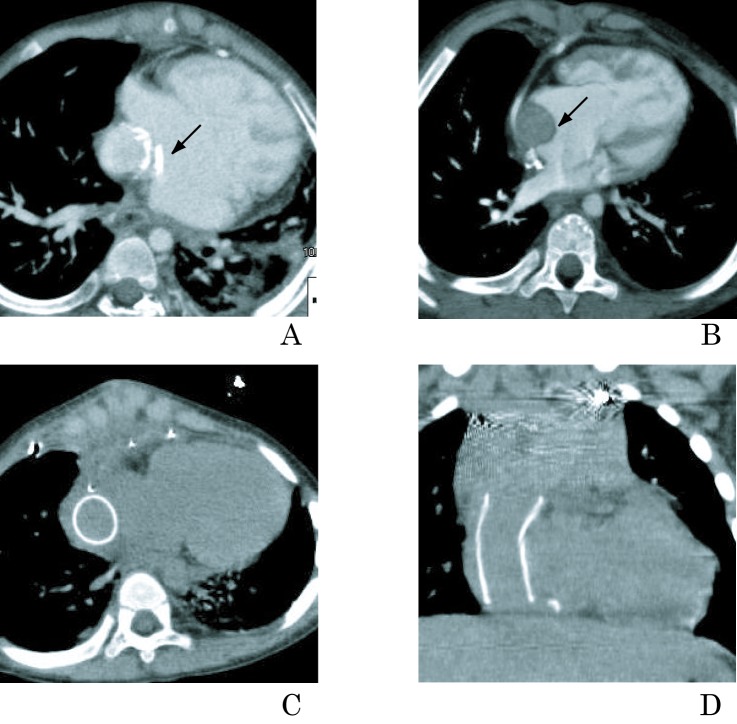
Multidetector-row computed tomographic views of polytetrafluoroethylene (PTFE) grafts used for atrial septal patches of Fontan procedure and extracardiac conduit of total cavopulmonary connection (TCPC). (A) Four-year-old girl with single ventricle who underwent lateral tunnel Fontan operation. Calcified intimal hyperplasia (arrow) is revealed on both sides of the atrial PTFE patch. (B) Four-year-old boy with DORV who had undergone lateral tunnel Fontan modification. Atrial septal patch (arrow) is clearly shown; no calcification is detected. Four-year-old boy with single ventricle who underwent extracardiac TCPC. Unenhanced MDCT was performed. The PTFE graft conduit is clearly shown with homogeneous high density in the axial (C) and coronal (D) views. *Reproduced with permission from American Heart Journal 2007; 153: 806.e1-8, Mosby, Inc.*

MDCT enabled the evaluation of prosthetic PTFE graft calcification. Our MDCT study revealed that 17% of ventricular septal defect (VSD) PTFE patches, 81% of PTFE in right ventricular outflow tract (RVOT) reconstruction (Fig. 4) and 25% of atrial septal patches of Fontan operation (Fig. 5) had calcified regions [9]. The limiting factor of PTFE conduits in RVOT is their relatively limited duration for one or more of the following reasons: patient outgrowth, calcification, thrombosis, thromboembolism, conduit obstruction, and valve regurgitation [21]. The postoperative interval in patients with calcification was significantly longer than that in those without calcification [9]. The calcified region gradually deteriorates after repair. The information acquired from MDCT images would be useful for balloon dilation and stent placement at this site. Thromboembolism is a significant contributor to late morbidity and mortality after the Fontan procedure [22, 23]. Foreign/prosthetic material placed within the heart can act as a nidus for thrombus formation, especially when the patch material is covered with pathologic intimal hyperplasia or calcification [22]. The atrial septal patches in our patients showed intimal hyperplasia with calcification. These findings could be an important evaluation for the prevention of thrombosis.

**Figure 6:**
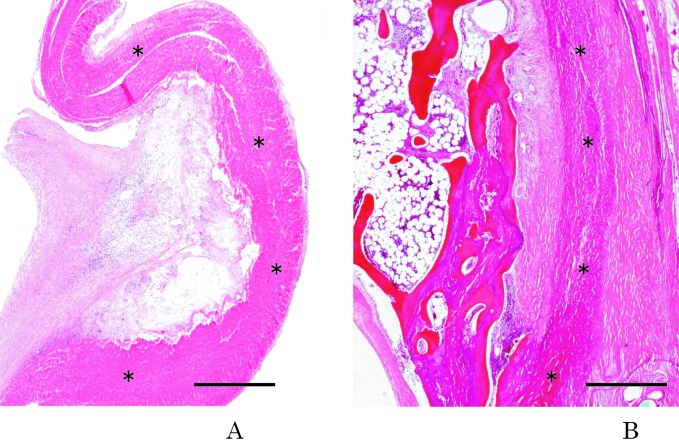
Histologic study. (A) Hematoxylin-eosin–stained section of PTFE (asterisk) obtained from the center of the graft after 2 years of implantation. Perigraft collagenous tissue infiltrates the microfibrous PTFE prosthetic wall. Hematoxylin-eosin stain indicates the presence of fibroblasts, collagen fibers, and neutrophils; the ingrowth of perigraft collagenous tissue in PTFE prostheses is revealed. Neovascularization is demonstrated in this area. The patient’s MDCT scan is shown in Figure 5B. (B) The PTFE patch layer is identified (asterisk); the prosthetic PTFE patch is encircled by excessive collagenous tissue. Bone formation with dystrophic calcification is visible on the left of the field: the bone formation appears eosinophilic, and the calcification appears basophilic. The patient’s MDCT scans are shown in Fiure 4C-F. Bar = 500 µm. *Reproduced with permission from American heart journal 2007; 153: 806.e1-8, Mosby, Inc.*

We examined the histopathological findings to investigate the relationship between calcification and the histological characteristics of prosthetic PTFE (Fig. 6). The grafts were explanted from patients undergoing reoperation. The MDCT findings were consistent with histological analysis in the evaluation of calcification. Calcification of patches, synthetic blood vessels, and other prostheses that is indistinct on conventional angiograms is clear on MDCT.

### Simultaneous observation of the anatomical relationships between arterial and venous system

**Figure 7:**
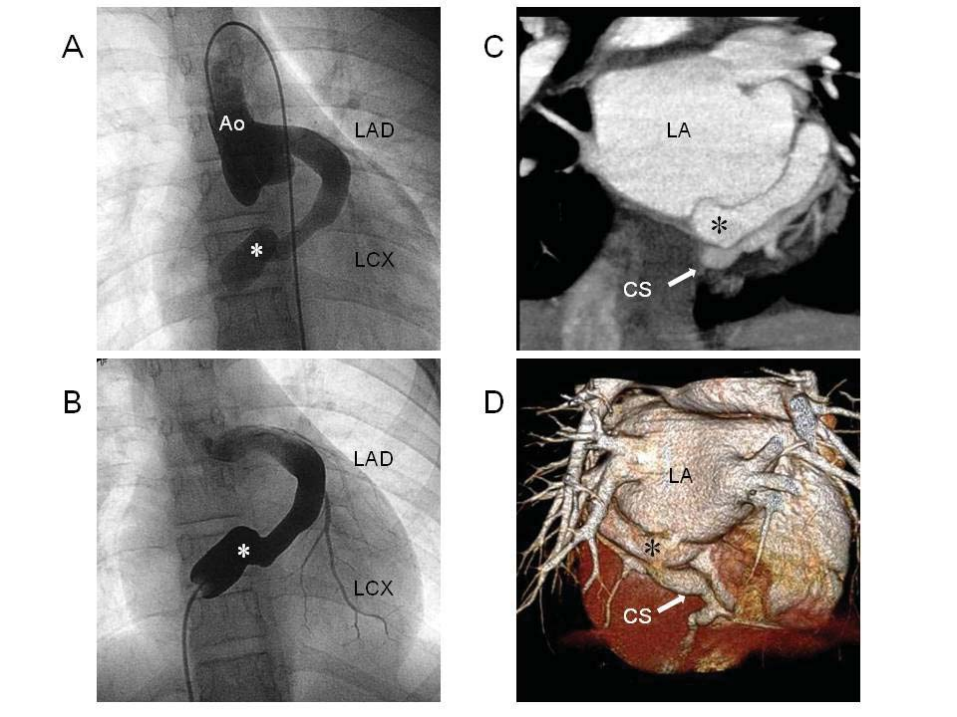
Coronary artery fistula in a 12-year-old girl. (A) Aortography shows an abnormal vessel draining into the right atrium. (B) Retrograde angiography shows that the left circumflex artery originated from this abnormally dilated vessel. (C) MDCT demonstrates that the coronary sinus is present next to this vessel. (D) A three-dimensional, volume-rendered image was useful for distinguishing the abnormal vessel from the coronary sinus. Ao, aorta; LAD, left anterior descending artery; LCX, left circumflex artery; LA, left atrium; CS, coronary sinus. *Reproduced with permission from Pediatric Cardiology 2010; 31: 168-169, Springer Science+Business Media, LLC.*

We presented the case of a 12-year-old girl with a coronary artery fistula [10]. The echocardiography and the aortography had shown the dilated left circumflex artery appeared to connect with the enlarged coronary sinus (Fig. 7A, B). It was quite difficult to distinguish the coronary sinus from the abnormal dilated vessel draining into the right atrium using echocardiography or conventional angiography. It was quite important and essential to make a precise diagnosis. If the left coronary artery is draining into coronary sinus and is forming the dilated vessel, the ligation of abnormal vessel adjacent to right atrium can be a contraindication, because the coronary circulation falls into failure.

MDCT images showed that the coronary sinus was present next to this abnormal vessel (Fig. 7C, D). This dilated vessel originated from left coronary artery and drained directly to the right atrium. MDCT can make a simultaneous observation of the anatomical relationships between arterial and venous systems on the same image, which can clarify whether abnormal blood vessels flow into atrium and whether neighboring veins are intact. In these cases, MDCT is a promising tool for precise depiction and determination of the optimal treatment.

### Compression of the airways by the great vessels

**Figure 8:**
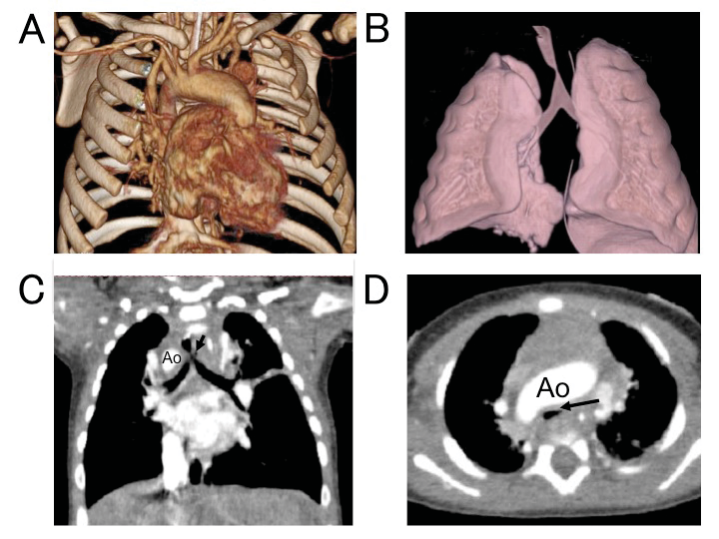
Tracheal compression by aortic arch in a 2-month-old child with corrected transposition of the great arteries (L-position), ventricular septal defect/pulmonary atresia. (A) 3D image in a patient with an right aortic arch. (B) 3D image of tracheal compression. Coronal (C) and horizontal (D) view of tracheal compression (arrow). *Reproduced with permission from Pediatric Radiology 2009;39:148-153, Springer Verlag.*

Children with CHD often experience preoperative and postoperative respiratory symptoms. The causes of airway obstruction in children might be related to extrinsic compression by vascular structures such as dilated branch pulmonary arteries, an aberrant innominate artery or a double aortic arch [24-26]. We reported that MDCT can demonstrate that normally formed right aortic arch also causes significant compression of the trachea, which results in airway obstruction (Fig. 8) [11].

**Figure 9:**
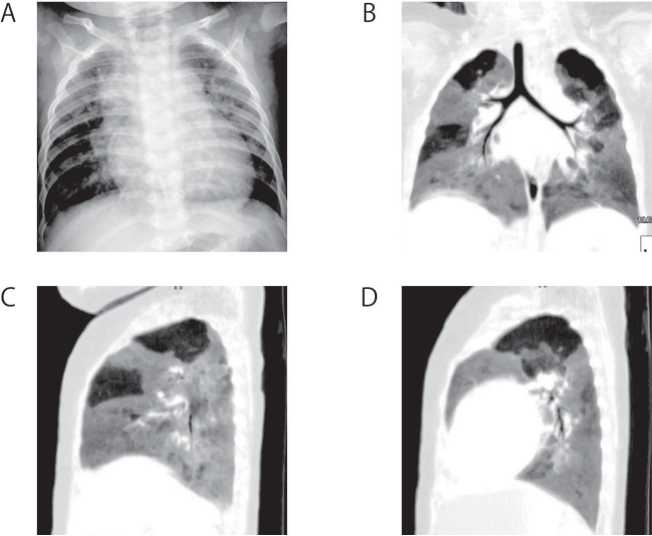
A 6-month-old girl with ventricular septal defect. Emphysematous change is not recognised with chest X-ray (A). Coronal view (B), sagittal view of right lung (C), and sagittal view of left lung (D) minimum-intensity projection imagings show that there are segmental emphysema in S1, 4 of right lung, and S1-2, 6, 7-8 of left lung. *Reproduced with permission from Heart, Lung and Circulation 2011, Elsevier limitied.*

Identifying tracheal compression using MDCT helps in the diagnosis of the cause of respiratory insufficiency. Furthermore, it would be helpful to be able to predict the likelihood of respiratory distress in patients with CHD. MDCT can replace traditional bronchography or bronchoscopy with a safer less-invasive method. MDCT images can provide valuable information because the entire airway can be viewed [27, 28]. Furthermore, we demonstrated that the segmental emphysematous change in children CHD associated with increased pulmonary blood flow can be evaluated using MDCT and minimum-intensity projection (minIP) imaging (Fig. 9).

## Discussion

In this review article, we indicated five pathological features, in which MDCT is useful not only as a non-invasive alternative to conventional angiography, but also as a tool for specific morphological diagnoses. (1) The patency of the peripheral pulmonary artery can be imaged and diagnosed using MDCT based on blood flow supplied from many small collateral vessels originating from the aorta, when Blalock-Taussig shunt total occlusion prevents catheter insertion into the artificial vessel and angiography is ruled out [7]. (2) Whereas pulmonary angiography and ultrasonography cannot accurately determine whether a coil extends to the left pulmonary artery, which can become constricted after coil embolization to treat patent ductus arteriosus, the location of the coil in the vessel can be accurately visualized by virtual endoscopy using MDCT [8]. (3) Calcification of patches, synthetic blood vessels, and other prostheses that is indistinct on conventional angiograms is clear on MDCT [9]. This information is useful for balloon dilatation and stent placement. (4) Simultaneous MDCT observations of the anatomical relationships between arterial and venous systems on the same image can clarify the detail diagnosis for surgical treatment [10]. (5) Compression of the airways by the great vessels can be diagnosed by MDCT [11]. Furthermore, segmental emphysematous change induced by peripheral pulmonary arterial dilatation can also be detected [12].

The issue of radiation exposure in children is extremely important. Children are more radiosensitive than adults to the same dose of organ radiation and because their life span is longer, the potential for radiation-induced malignancies to develop is higher [29, 30]. It is important to reduce radiation dose to as low as reasonably achievable (ALARA principle) [31, 32]. In this respect, we have to select the children with cardiovascular condition suitable for the MDCT examination among the patients with various congenital heart diseases.

MDCT is useful not only as a non-invasive alternative to conventional angiography, but also as a tool which has unique feature for specific morphological diagnoses. In the future, it will be necessary to accumulate experience in the recognition of pathological conditions under which MDCT is necessary and to perform the appropriate tests.
